# Modeling Desorption Rates and Background Concentrations of Heavy Metals Using a One-Dimensional Approach

**DOI:** 10.3390/toxics13060421

**Published:** 2025-05-22

**Authors:** Wendy Tatiana Gonzalez Cano, Serguei Lonin, Kyoungrean Kim

**Affiliations:** 1 Marine Environmental Research Department, Korea Institute of Ocean Science and Technology, Busan 49111, Republic of Korea; tatiana@kiost.ac.kr; 2 KIOST School, University of Science and Technology (UST), Busan 49111, Republic of Korea; 3 Admiral Padilla Naval Academy of Cadets, Isla Naval Manzanillo, Cartagena de Indias 130001, Colombia; oceanmet.ltda@yahoo.com

**Keywords:** estuarine sediments, heavy metals, background concentrations, desorption rate, cohesive sediment transport, mathematical modeling

## Abstract

Harmful heavy metals (HHMs) in marine sediments pose significant ecological and human health risks. This research developed a novel one-dimensional mathematical model to investigate the desorption rates and background concentrations ($C_{bg}$) of HHMs in cohesive sediments of coastal environments, using Cartagena Bay (CB), Colombia, as a reference for estuarine systems. The model integrates mass balance and molecular diffusion equations incorporating porosity and tortuosity. Both the particulate and dissolved phases of HHMs were considered. Numerical experiments were conducted over 28 years with a daily time step, simulating four primary hydrodynamic processes: molecular diffusion, desorption, sedimentation, and turbulent water exchange. The spatiotemporal evolution of $\;C_{bg}$ provides valuable insights for sediment modeling, policy development, and advancing the understanding of HHM pollution in sediments. Results of the model align closely with empirical data from CB, demonstrating its applicability not only to local conditions but also to similar contaminated areas through a generalized approach. This model can be used as a reliable computational tool for managing coastal environments.

## 1. Introduction

Heavy metal pollution poses a global environmental concern due to its severe toxicity [1,2], long-term persistence, and bioaccumulation in food chains [3,4,5]. Harmful heavy metals (HHMs) are continuously introduced into the environment [6] through natural and anthropogenic sources [7,8]. In estuarine waters, the presence of HHMs is generally observed in two distinct phases: dissolved in the water column and particulate adsorbed on the sediments. The partitioning of HHMs between these phases depends on the physical and chemical characteristics of suspended particles [9,10], in conjunction with environmental conditions such as salinity, pH, and dissolved organic matter [10].

Upon entering surface waters, HHMs are transported by rivers through wash load transport and eventually accumulate in marine sediments. Hydro-sedimentary processes such as desorption [11], resuspension, and dredging can release these contaminants back into the overlying water column [12], affecting water quality, the marine environment, and human health. Although HHM adsorption initially occurs near areas with significant anthropogenic activities, this study emphasizes the downstream consequences, particularly focusing on sediment contamination.

Various researchers rely on predicting interactions between water and sediments as a critical method for understanding HHM pollution [5,13,14,15]. Mathematical models have become powerful tools to address complex research questions related to coastal environments, offering reliable, cost-effective, and time-saving approaches [16].

Specifically, reaction-transport models, as described by Boudreau [17], Lynch and Officer [18], and Nicolis [19], have been pivotal in advancing sediment diagenesis and biogeochemical modeling, integrating key processes such as molecular diffusion, advection, and chemical reactions. These frameworks form the theoretical foundation of this study, focused on the vertical distribution and temporal evolution of HHMs in sediments.

Previous studies, such as Wu et al. [10], developed a two-dimensional (2D) transport model, later integrated into a one-dimensional (1D) framework to simulate the movement of dissolved and particulate HHMs along estuaries. However, these models did not explicitly address the accumulation of HHM contaminants or their subsequent phase evolution within the substrate, a significant gap in understanding the long-term impacts and interactions of HHMs with sediment dynamics.

Numerous studies have focused on HHM accumulation in sediments near contamination sources. However, limited studies have been published on desorption rates and their dynamics in downstream depositional environments such as lakes, lagoons, and estuaries. In systems dominated by wash load transport of fine cohesive sediments, the deposition and accumulation pathways of metal-bearing particles are not well documented. Apparently, sediment accumulation may occur slowly in distal areas, comparable to sorption rates, or rapidly near river mouths due to abrupt precipitation of materials, limiting metal exchange with the water column. These spatial heterogeneities affect HHM redistribution within sediments, complicating the estimation of background concentrations $\left(C_{bg\;}\right)$.

This study advances existing frameworks by proposing a novel 1D model that couples transport and reaction processes. This model is designed for application at each computational node of a generalized three-dimensional (3D) hydrodynamic model, serving as a boundary condition at the water–sediment interface to simulate HHM accumulation and sediment evolution over time. The innovation of this approach is the integration of both dissolved and particulate HHM phases. Four critical hydrodynamic processes are quantified and modeled to evaluate their influence on HHM dynamics. This model, in accordance with empirical data, assumes that the dissolved-phase concentration ($C_{d}$) is considerably lower than the particulate-phase concentration ($C_{p}$) in the water column ($C_{d}$ *<<* $C_{p}$).

To demonstrate the model’s applicability, simulations were referenced against estuarine conditions in the Cartagena Bay, Colombia (CB), a system subject to intense sedimentation and HHM inputs. Results obtained through this model align closely with empirical observations, reinforcing its validity.

The aim of this study is (1) to develop a novel 1D mathematical model to investigate HHM dynamics in estuarine sediments and (2) to elucidate the processes of HHMs governing background concentrations ($C_{bg}$). These concentrations serve as critical indicators for identifying anthropogenic inputs [20] and facilitate the formulation of effective management and remediation strategies [7,21]. This research represents the first attempt to establish the $\;C_{bg}\;$ of HHMs in Colombia, highlighting its significance in addressing local and regional environmental concerns.

The model is applicable beyond the specific conditions of the Colombian coastline and could be effectively extended to various aquatic systems, including rivers, estuaries, and lakes worldwide affected by sediment contamination. Model outputs, including dynamic profiles illustrating the temporal drift of $C_{bg}$, are presented and critically discussed.

## 2. Materials and Methods

### 2.1. Cartagena Bay: A Reference System for Estuarine Conditions

**Figure 1:**
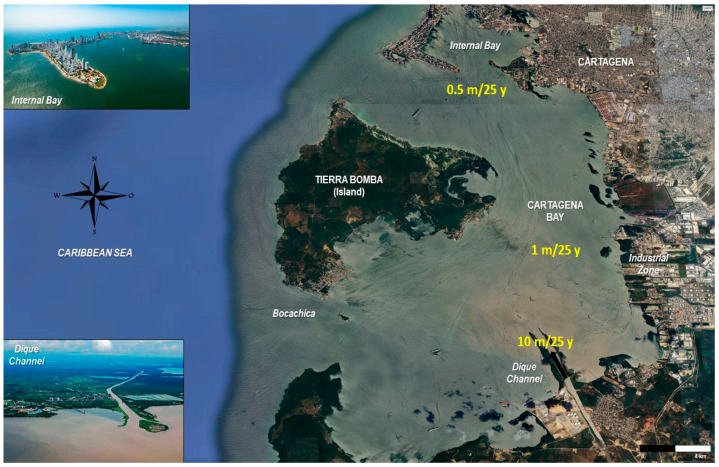
Location of the Cartagena Bay, Colombia (10°24′ N, 75°30′ W), showing the variability of sedimentation across the bay with three estimated deposition rates: Dique Channel mouth: 10 m/25 y; the central bay: 1 m/25 y; and the northern sector: 0.5 m/25 y. The Dique Channel is highlighted as the main sediment input pathway. Source: authors, modified from Google Earth; pictures taken from Google Images (accessed May 2025).

CB is a semi-enclosed estuarine system on Colombia’s Caribbean coast (Figure 1) (10°16′–10°26′ N, 75°30′–75°35′ W), with an average depth of 16 m, a maximum depth of 32 m, and a surface area of 84 km^2^ [22]. The bay receives large amounts of sediments [23], nutrients, wastewater runoff [24], and contaminants from the Dique Channel [25,26], an artificial structure connected to the extensive Magdalena River basin (260,000-km^2^) [26,27].

The Magdalena River transports a sediment flux of 184 Mt yr^−1^ and delivers the highest freshwater discharge (6496 m^3^ s^−1^) and sediment load (144 Mt yr^−1^) to the Caribbean [26,27]. Seasonal rainfall from the Magdalena River, where the Dique Channel diverges, strongly influences the hydrology and sediment quality of CB [28]. CB’s sedimentation patterns and morpho-dynamic characteristics have been previously studied through observations and modeling [29,30,31]. Due to wash load transport, HHM adsorption does not occur in CB but rather in distant sources before HHMs are transported downstream. This explains the HHM accumulation in precipitated sediments.

### 2.2. Mathematical Model

#### 2.2.1. Definition of the Physical Problem

This research considers the wash load transport [32] of fine cohesive sediments (silt and clay) loaded with HHMs in particulate form. The primary source of HHMs is the rapid upstream industrialization in the Magdalena River. Particles are deposited at the bottom in the form of flocs. In the porous medium of precipitated sediments, desorption continues but at a lower rate than that during transport in the water column.

In the water column, HHMs exist in colloidal, particulate, and dissolved phases. The concentration of HHMs in dissolved form ($C_{d}$) is generally lower than that in particulate form ($C_{p}$). For instance, field measurements in the Magdalena River show that $C_{d}$ values are approximately 1000 times lower than $C_{p}\;$ in suspended and bottom sediments. This aligns with observations by Bartlett and Craig (1981) [33], who reported strong correlations (r = 0.94) between mercury (Hg) and silt in British estuaries, demonstrating Hg’s affinity for fine particles and sulfur-rich organic matter (<0.0625 mm). The volumetric concentration of suspended sediments at the transition between dilute and concentrated systems is typically below 10^−4^ (dimensionless). Considering that desorption occurs over several years (~6 years), the mass of HHMs released from particulates is dispersed into a much larger water volume, further supporting the assumption $C_{d}\;$<< $C_{p}$. Combined with the infinite-dilution diffusion concept [34], these theoretical and empirical insights justify the assumption as a valid simplification within this modeling framework.

Hereafter, we assume that $C_{d}$ concentrations are multiplied by the constant *K_d_*, which represents the equilibrium distribution coefficient. In the sediment substrate, these concentrations are also assumed to reach equilibrium due to limitations in molecular diffusion, which is partially restricted by porosity (*n*) and tortuosity (θ). Lower porosity and higher tortuosity restrict molecular diffusion, reducing HHM exchange with overlying waters and consequently promoting high accumulation and persistence within the sediment layer.

Tortuosity quantifies the complexity of pore pathways through which water and dissolved substances, such as HHMs, move within sediment layers [35]. Porosity, defined as the ratio of pore volume to total sediment volume [17], also plays a critical role in transport dynamics. Lower porosity implies fewer and smaller pores, restricting mobility and facilitating contaminant accumulation. As sediment compacts over time, porosity typically decreases with depth (*z*), becoming a time-dependent function. This leads to a gradual increase in substrate thickness in the absence of resuspension.

The desorption rate $\left(\gamma \right)$, reflects the release of HHMs from $C_{p}\;$ to $C_{d}$ and depends on the grain diameter of sediments ($d_{50}$), their porosity (*n*), salinity (*S*), and pH. The porosity and tortuosity together influence molecular diffusion, calculated in the model using the Schmidt number (*Sc*), a dimensionless parameter used to characterize the relationship between the molecular viscosity of water and the diffusion of substances [36].

**Figure 2:**
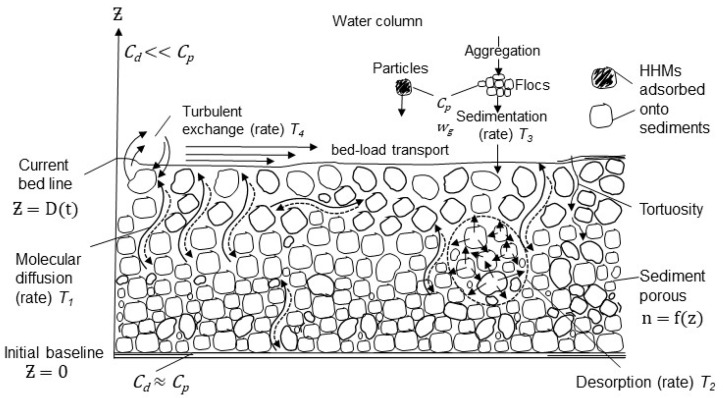
Conceptual model of HHM dynamics at the water–sediment interface and within the sediment substrate.

A 1D vertical model was formulated, neglecting the horizontal dispersion of HHMs, with the vertical axis directed upward from *z* = 0 (the reference level is assumed to be the starting point of sedimentation, as shown in Figure 2). The 1D model can be considered a sufficient approximation, considering that (a) the relationship between the vertical scale of the sediment layer and its horizontal extent along an estuary or river is small, and (b) exchange processes in the substrate in the vertical direction are much faster than the horizontal dynamics.

The domain is defined as $\left\{0\leq z\leq D\left(t\right);t\geq 0\right\}$, where *D* = sediment thickness as a function of time (*t*). At the initial time, *D* was set as *D*(*t* = 0) = 0. To avoid singularities when solving the differential equations of the model, we assumed that $\frac{\partial D}{\partial t}>0,\forall t\;$ under the absence of resuspension. The dynamics of the layer *D*(*t*) are expressed as follows:

(1) $$\frac{\partial D}{\partial t}={-w}_{g}C_{v}{\left(1-n\right)}^{-1}=\frac{{-w}_{g}{\left(1-n\right)}^{-1}C_{m}}{\rho_{S}},\;$$

where $w_{g}\;$ is the settling velocity of sediments due to gravity, given by the Stokes formula ($w_{g}<0$), and $C_{v}$, $C_{m}$, and $\rho_{s}$ are the volumetric and mass concentrations of suspended sediments and their density, respectively. Within the bottom substrate, the molecular diffusion flux of the $C_{d}$ is defined as follows:

(2) $$Q=\alpha_{S}\nu \frac{\partial C_{d}}{\partial z},$$

where ν is the kinematic molecular viscosity of water (Constant) and $\alpha_{S}$ defines the inverse Schmidt number ($\alpha_{S}={Sc}^{-1}$) [36], which generally depends on time and substrate level or porosity *n.*

To determine the desorption rate $\gamma =f\left(d_{50},n,S,pH\right)$, at least three timescales must be considered: (1) the molecular diffusion rate (*T*_1_) of HHMs; (2) the desorption rate (*T*_2_); and (3) the sediment deposition rate (*T*_3_) at the bottom, as follows:

(3) $$T_{1}=\frac{D_{0}^{2}Sc}{\nu },$$

(4) $$T_{2}=\frac{1}{2\gamma },$$

(5) $$T_{3}=\frac{D_{0}}{w_{g}C_{v}}.$$

This study has focused on the formulation and evaluation of a generalized 1D model for simulating HHM behavior in estuarine sediments, rather than on site-specific applications. The parameters of the model, particularly sedimentation and desorption rates, were calibrated within observed HHM concentrations reported in empirical data from CB [24,25,27,37]. The mathematical model was implemented and numerically solved using FORTRAN 90. Model outputs were compared to published sediment data from CB [23,24,25,38]. Simulated $C_{bg\;}$ ranging from 1.0–2.4 mg kg^−1^ dw closely matched these empirical values at the sediment base (*z* = 0). The median grain diameter (*d*_50_) (Table 1) was measured using a laser diffraction particle size analyzer. HHM concentrations were determined in the collected data using standard laboratory procedures involving acid digestion followed by quantification via atomic absorption spectrometry (AAS) or inductively coupled plasma mass spectrometry (ICP-MS). Although full calibration was limited by data availability, alignment with observed depth-integrated values provides partial validation.

Four numerical simulation scenarios (Cases 1–4) were analyzed to investigate the influence of hydrodynamic parameters on HHM accumulation dynamics. Case 1 (γ = 5 × 10^−8^ s^−1^) represents relatively fast desorption conditions, whereas Case 2 (γ = 10^−8^ s^−1^) examines the system response under slower desorption dynamics. Case 3 simulates a time-dependent increase in the $C_{p}$ of HHMs, linearly increasing from 0 to 2.4 mg kg^−1^ over 28 years, reflecting observed historical contamination trends from distant sources such as the Magdalena River. Case 4 incorporates variable sediment inputs (55–250 m^3^ s^−1^), modeled through stochastic annual fluctuations to replicate seasonal variations typical of the Dique Channel. These scenarios evaluate sedimentation and desorption processes under contrasting environmental conditions with broader applicability.

#### 2.2.2. Governing Equations and Boundary Conditions

The mathematical formulation of the problem is expressed in Equation (1). The governing equations for $C_{p}$ and $C_{d}$ of HHMs are defined clearly below (Equations (6) and (7)), including mass balance constraints and desorption processes:

(6) $$\frac{\partial C_{p}}{\partial t}=-\gamma \left(C_{p}-C_{d}\right)-\frac{w_{g}}{D}C_{p0}\delta \left(z-D\right)$$

(7) $$\frac{\partial C_{d}}{\partial t}=\gamma \left(C_{p}-C_{d}\right)+\frac{\partial }{\partial z}\left(\alpha_{S}\nu \frac{\partial C_{d}}{\partial z}\right),$$

(8) $${AC}_{d}+\alpha_{S}\nu \frac{\partial C_{d}}{\partial z}=0,\;\mathrm{at}\;z=D(t)$$

(9) $$\frac{\partial C_{d}}{\partial z}=0,\;\mathrm{at}\;z=0$$

In Equation (8), *A* is a constant defined in Appendix B, based on the fact that in the water column, $C_{d}\ll C_{p}$ due to diffusion in open water systems. As stated by the no-flux condition in Equation (9), an asymptotic equilibrium is assumed at *z* = 0 between $C_{p}$ and $C_{d}$, with values equal to the $C_{bg}\;$ to be defined in this study.

This boundary condition assumes equilibrium at lower sediment layers (*z* = 0), reaching a balance due to decreased porosity and restricted molecular diffusion over time. In Equation (9), the molecular flux of $C_{d}$ at *z* = 0 is assumed to be zero. The boundary conditions at *z* = 0 and *z* = *D*(*t*) ensure the mass balance of HHMs within the sediment, accurately representing fluxes and the conservation of mass. At *z* = *D*(*t*), the boundary condition models the exchange between the sediment and the overlying water column.

#### 2.2.3. Numerical Solution Under Variable Boundary Conditions

Equations (6)–(9), with their respective initial conditions of $C_{p}\left(z,\;t=0\right)=C_{d}\left(z,t=0\right)=0$, have a variable boundary at an initial thickness of *D*(*t* = 0) = 0. The vertical coordinate (*z*) was transformed into a non-dimensional coordinate, following Yao et al. [13], to improve numerical solution robustness. The system herein was reformulated using a new variable:

$$y=\frac{z}{D\left(t\right)}$$

This becomes $y_{j}=\left(j-1\right)∆y$; j=1, …, *N*;$\;∆y=\frac{1}{N-1}$, where *N* is the number of vertical computational nodes. Thus, combining Equations (6) and (7) with Equation (1), we obtain the following:

(10) $$\frac{\partial C_{p}}{\partial t}+\frac{y}{D}\frac{w_{g}C_{V}}{\left(1-n\right)}\frac{\partial C_{p}}{\partial y}=-\gamma \left(C_{p}-C_{d}\right)-\frac{w_{g}}{D}C_{p0}\delta \left(z-D\right),$$

(11) $$\frac{\partial C_{d}}{\partial t}+\frac{y}{D}\frac{w_{g}C_{V}}{\left(1-n\right)}\frac{\partial C_{d}}{\partial y}=\gamma \left(C_{p}-C_{d}\right)+\frac{\nu }{D^{2}}\frac{\partial }{\partial y}\left(\alpha_{S}\frac{\partial C_{d}}{\partial y}\right).$$

These equations were then discretized using an implicit time scheme, ensuring numerical stability regardless of the sediment thickness, *D*. The first derivatives concerning y were then represented using an “upward” scheme of ${O(∆y}^{1})$. The solution was obtained using the Thomas factorization algorithm.

## 3. Results

### 3.1. Estimation of Molecular Diffusion (T_1_), Desorption (T_2_), and Sedimentation (T_3_)

To estimate the timescales (*T*_1_, *T*_2_, *T*_3_) given by Equations (3)–(5), a characteristic sediment thickness ($D_{0}\;$ = 1 m), molecular viscosity (*v* = $10^{-6}$ m^2^ s^−1^), and Schmidt number (*Sc* = 100) were adopted [6]. With these parameters, *T*_1_ was estimated to be 3 years. For *Sc* = 10, *T*_1_ was approximately 115 days. According to Liu et al. [42], the values of γ vary between 10^−8^ and 10^−9^ s^−1^. For γ= 5 × 10^−8^ s^−1^, the timescale of *T*_2_ was 3.15 years.

Finally, assuming that the volumetric concentration was between 10^−4^ and 10^−5^ and the settling velocity due to gravity was 10^−5^ m/s, the value of *T*_3_ was greater than 31 years. Therefore, *T*_3_ >> max (*T*_1_, *T*_2_) *T*_3_ has the slowest timescale, while the other two timescales were similar to each other.

In addition to previously defined timescales (*T*_1_–*T*_3_), a fourth timescale (*T*_4_), representing the turbulent water column exchange of dissolved HHMs at the sediment–water interface, was determined. Resuspension was not considered, as particulate-bound HHMs do not significantly participate in this exchange.

### 3.2. Numerical Experiments

**Figure 3:**
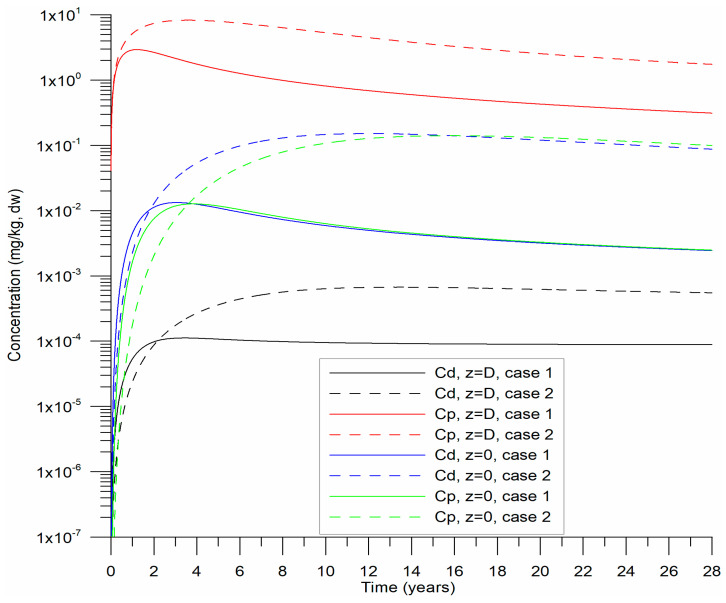
Temporal evolution of particulate ($C_{p}$) and dissolved ($C_{d}$) HHM concentrations at the variable bed level *D*(*t*) and the basal level (*z* = 0) of the bottom substrate for Cases 1 and 2.

**Figure 4:**
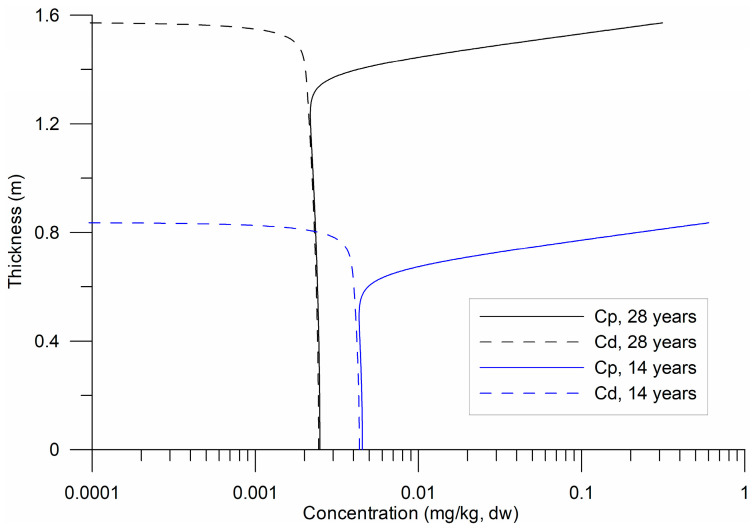
Temporal variability in the vertical profiles of particulate ($C_{p}$) and dissolved ($C_{d}$) HHMs for 14 and 28 years of sedimentation (γ= 5 × 10^−8^ s^−1^).

The sediment density ($\rho_{s})$ was set at 2650 kg m^−3^, with porosity (*n*) fixed at 0.4. A drag coefficient ($C_{D}$) of 2 × 10^−3^ [39] and an initial dynamic velocity of 0.01 m s^−1^ were applied. Numerical experiments were conducted over 28 years, using a daily time step (1 d). Figure 3 illustrates the temporal evolution of $C_{d}$ and $C_{p}$ from the beginning of precipitation on both the surface and base when the desorption coefficient γ is varied. Sedimentation was assumed to continue uniformly over the 28-year simulation at constant rates, with fixed HHM concentrations in the precipitated sediments. In Figure 4, profiles of HHM concentrations in sediments are presented at both the midpoint and the end of the numerical experiments.

Over 28 years, the sediment layer grew to 1.6 m, which aligned well with data from CB and served as a reference for this study. Following an initial transient period (Figure 3), the $C_{d}$ and $C_{p}$ stabilized. The desorption rate was slower, corresponding to 6.3 years on the timescale of this process, compared to the reference value of 3.15 years.

The vertical profiles exhibited an exponential variation in the upper layer of the substrate (Figure 4), over 30–40 cm, followed by a uniform distribution. The variation was attributed to the vertical molecular diffusion of HHMs and their loss, particularly in $C_{d}$, due to turbulent exchange with the water column at the bottom. The uniform distribution in the lower sublayer indicates equilibrium between the two phases; however, this equilibrium was not constant (Figure 4). Equilibrium stability between $C_{d}$ and $C_{p}$ is crucial for ecological risk assessments, as it governs HHM bioavailability and potential toxicity in benthic ecosystems [43].

## 4. Discussion

### 4.1. Temporal Evolution of Background Concentrations Estimated by the Model

**Figure 5:**
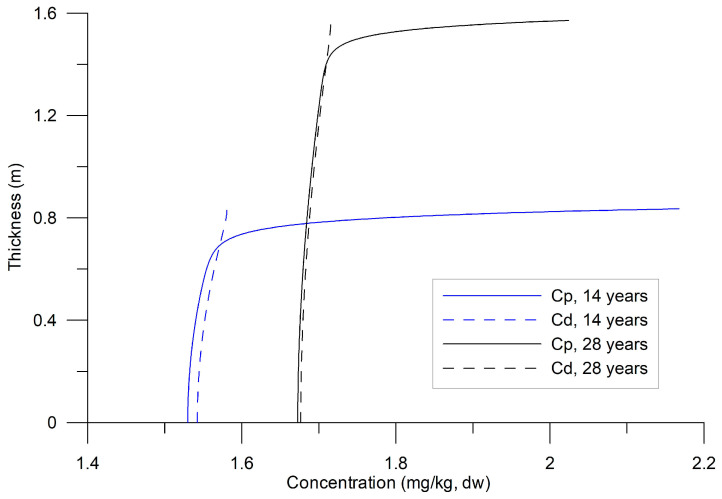
HHM concentrations in sediments under conditions of limited exchange between the dissolved phase and the water column.

A drift value, tentatively called the background $C_{bg}$, was observed, characterized by a gradual decrease over time. This decrease is attributable to continuous slow molecular diffusion within the non-zero sediment porosity, transporting HHMs towards the sediment–water interface. Subsequent experiments were conducted by minimizing the turbulent exchange of the $C_{d}$ with the water column above the bed. This occurs when $u_{*}\to 0$ (Figure 5). A nearly uniform distribution of $C_{d}$ in the vertical direction was observed, along with the input of $C_{p}$ at the bottom surface. The total concentrations of HHMs in the sediments were the sum of $C_{p}$ and $C_{d}$/$K_{d}$, and in the laboratory, a single value was defined: “HHM concentrations in sediments”.

**Figure 6:**
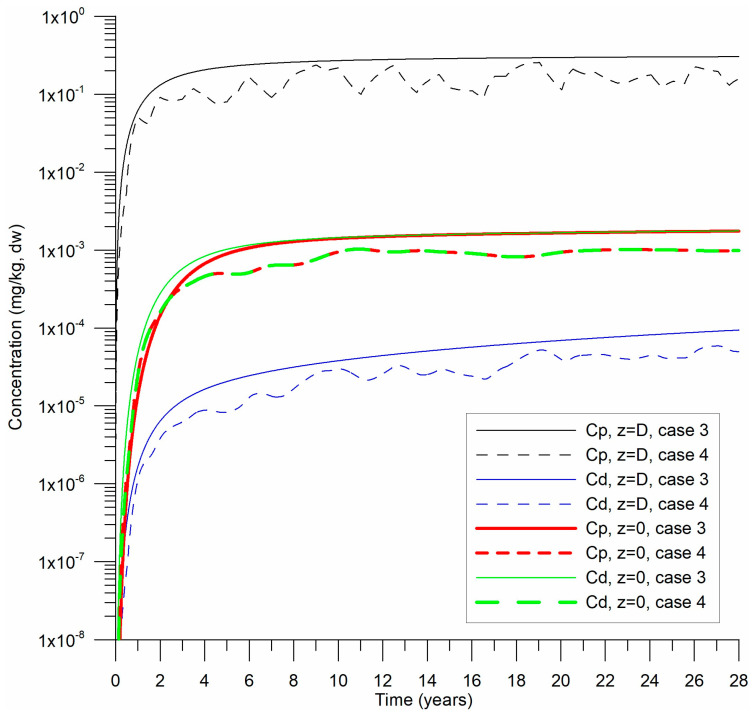
Temporal evolution of particulate ($C_{p}$) and dissolved ($C_{d}$) HHM concentrations at the variable bed level *D*(*t*) and at the basal level (*z* = 0) of the bottom substrate for Cases 3 and 4.

For Case 1, γ was set to 5 × 10^−8^ s^−1^, which suggested a relatively faster desorption rate compared to Case 2 (γ= 10^−8^ s^−1^). Two additional cases (Cases 3 and 4) were simulated (Figure 6). In Case 3, the initial particulate concentration $C_{p0},\;$ increased linearly from 0 to 2.4 mg kg^−1^ over 28 years, reflecting observed trends in CB associated with increased HHM loading from a distant source, the Magdalena River. Case 4 was similar to Case 3, but with a variable sediment input that varied between 55 and 250 m^3^ s^−1^ to replicate the Dique Channel’s seasonal flow, using annually periodic white-noise perturbations (stochastic values 0–1).

The cases in Figure 6 were compared to Case 1 in Figure 3 where HHMs in sediments accumulated more slowly. Notably, when HHM loading gradually increased (Case 3), the concentrations at the sediment base (*z* = 0) consistently reached equilibrium ($C_{d}$ = $C_{p}$ = $C_{bg}$). Conversely, seasonal variations in sediment load from the Dique Channel (Case 4) did not significantly alter the equilibrium $C_{bg}\;$ value. These findings imply that $C_{bg}$ values remain stable despite short-term fluctuations, highlighting their value as robust indicators for long-term ecological risk assessments.

### 4.2. Dimensionless Analysis and HHM Dynamics

To perform an analysis of the systems in Equations (1) and (6)–(9), dimensionless variables were introduced as follows:

$${\tilde{C}}_{d}=\frac{C_{d}}{C_{bg}};{\tilde{C}}_{p}=\frac{C_{p}}{C_{bg}};\;{\tilde{t}}_{1}=\frac{t}{T_{1}};{\tilde{t}}_{2}=\frac{t}{T_{2}};{\tilde{t}}_{3}=\frac{t}{T_{3}};\tilde{z}=\frac{z}{D};\;\frac{{\tilde{t}}_{2}}{{\tilde{t}}_{1}}=\frac{2\gamma D^{2}}{\alpha_{S}\nu }=a;\;\;{\tilde{C}}_{p0}=\frac{C_{p0}}{C_{bg}};\frac{{\tilde{t}}_{1}}{{\tilde{t}}_{3}}=\frac{w_{g}D}{\alpha_{S}\nu }=\beta ;\;\tilde{D}=\frac{D}{D_{0}};\;\zeta =\frac{\alpha_{S}\nu }{u_{*}D};\;{\tilde{C}}_{p0}=\frac{C_{p0}}{C_{bg}}.$$

The “~” symbol implies a dimensionless variable.

The systems originally presented in Equations (6) and (7), along with their respective conditions (8) and (9), are reformulated in dimensionless form and have been renumbered consecutively as Equations (12)–(15), as follows:

(12) $$\frac{\partial {\tilde{C}}_{p}}{\partial {\tilde{t}}_{1}}=-a\left({\tilde{C}}_{p}-{\tilde{C}}_{d}\right)-\beta {\tilde{C}}_{p0}\delta \left(\tilde{z}-\tilde{D}\right),$$

(13) $$\frac{\partial {\tilde{C}}_{d}}{\partial {\tilde{t}}_{1}}=a\left({\tilde{C}}_{p}-{\tilde{C}}_{d}\right)+\frac{\partial }{\partial \tilde{z}}\left({\tilde{\alpha }}_{S}\frac{\partial {\tilde{C}}_{d}}{\partial \tilde{z}}\right),$$

(14) $${C_{D}^{\frac{1}{2}}\tilde{C}}_{d}+\zeta \frac{\partial {\tilde{C}}_{d}}{\partial \tilde{z}}=0,\;at\;\tilde{z}=\tilde{D}$$

(15) $$\frac{\partial {\tilde{C}}_{d}}{\partial \tilde{z}}=0,\;at\;\tilde{z}=0.$$

Adding Systems (12) and (13) and using conditions (14) and (15), the results are as follows:

(16) $$\int_{0}^{\tilde{D}}\frac{\partial {(\tilde{C}}_{p}+{\tilde{C}}_{d})}{\partial {\tilde{t}}_{1}}d\tilde{z}=\left|\beta \right|{\tilde{C}}_{p0}-\frac{{C_{D}^{\frac{1}{2}}\tilde{C}}_{d}\left(\tilde{z}=\tilde{D}\right)}{\zeta }.$$

Applying Leibniz’s rule and reformulating Equation (1) in terms of “fast” time ${\tilde{t}}_{1}$, we obtain the following:

(17) $$\frac{\partial \tilde{D}}{\partial {\tilde{t}}_{1}}=\frac{C_{v}}{\left\{\left|\beta \right|\left(1-n\right)\right\}}$$

The temporal variation in the total HHM concentration of sediments over the entire extent of its layers can be defined by the following equation:

(18) $$\frac{\partial }{\partial {\tilde{t}}_{1}}\int_{0}^{\tilde{D}}{(\tilde{C}}_{p}+{\tilde{C}}_{d})d\tilde{z}=\left|\beta \right|{\tilde{C}}_{p0}-\frac{{C_{D}^{\frac{1}{2}}\tilde{C}}_{d}\left(\tilde{z}=\tilde{D}\right)}{\zeta }+\frac{C_{v}}{\left\{\left|\beta \right|\left(1-n\right)\right\}{\left.{(\tilde{C}}_{p}+{\tilde{C}}_{d})\right|}_{\tilde{D}}}.$$

The first term on the right-hand side of Equation (18) represents the input of HHMs in $C_{p}$ into the sediment column, while the second term accounts for their loss through exchange with the overlying water in the $C_{d}$. The final term corresponds to the increase in the total HHM concentration due to changes in substrate thickness and its redistribution in the column. This term was considered less relevant when the *T*_3_ scale represented a slow time relative to *T*_1_ and *T*_2_. Within the same body of water, as exemplified by CB, the *T*_3_ scale is spatially variable.

If $\frac{\partial \tilde{D}}{\partial {\tilde{t}}_{1}}\approx 0$ for the “fast” time in System (17), then systems (12)–(15) are represented as parabolic equations whose asymptotes in time are ${\tilde{C}}_{p}={\tilde{C}}_{d}$. Steady-state conditions are possible only if the particle sedimentation process stops.

For →∞, $\frac{\partial }{\partial \tilde{z}}\left({\tilde{\alpha }}_{S}\frac{\partial {\tilde{C}}_{d}}{\partial \tilde{z}}\right)=0$, considering condition (15) at a given level, the molecular flow is equal to zero throughout the substrate.

In this case, ${\tilde{C}}_{p}={\tilde{C}}_{d}=1\forall t$ (background concentration). The only reason this did not occur throughout the entire sediment column is the permanent entry of HHMs, owing to their precipitation on particles at the bottom and the exchange of the diluted phase with the water column at the same vertical level.

The simulated sedimentation rates ranged from 0.5 m per 25 years (low deposition) to 10 m per 25 years (at the river mouth). Figure 1 presents the variability in sedimentation across CB, highlighting three depositional zones: Dique Channel mouth: 10 m/25 y; the central bay: 1 m/25 y; and the northern sector: 0.5 m/25 y. Therefore, the 1D model should be applied at each calculation node of a 3D hydrodynamic mesh, with local scales adjusted accordingly.

The universality of the proposed model lies in its formulation using dimensionless variables and scale parameters. The analysis of dimensionless equations (Equations (12)–(15)) allows for a broad spectrum of environmental conditions. These ranges reflect both the intra-basin variability within CB, such as differences between river mouth and inner-bay sedimentation rates, and potential conditions in other estuarine systems. This dimensional analysis enables the model to be applied across geographically distinct water bodies, provided that the local sedimentation dynamics and hydro-sedimentary conditions are within comparable parameter bounds.

Considering the timescale variation in the main processes, the desorption rate, the average speed of sediment settling by gravity, and the molecular viscosity of water were fixed. The thickness of the sediment layer and its porosity (through the Schmidt number) varied within reasonable limits, characterizing CB as an example of an estuary. The resulting values of the dimensionless parameters for systems (12)–(15) were a = 10^−3^ to 10^3^; β = 10^2^ to 10^4^; ζ/$C_{D}^{\frac{1}{2}}$ = 0.25 (10^−1^–10^−5^); and ${\tilde{\alpha }}_{S}$ = 10^−2^ to 10^2^.

Under these conditions, Equations (12) and (13) can present multiple scenarios of HHM dynamics because the ratios of scale $\frac{{\tilde{t}}_{2}}{{\tilde{t}}_{1}}$ and $\frac{{\tilde{t}}_{1}}{{\tilde{t}}_{3}}$ change four to six orders of magnitude. In the case where the parameter a = $\frac{{\tilde{t}}_{2}}{{\tilde{t}}_{1}}$, the scales become inverted. Regarding condition (14), the relationship ζ/$C_{D}^{\frac{1}{2}}$<< 1 implied an abrupt gradient $\frac{\partial {\tilde{C}}_{d}}{\partial \tilde{z}}$, which was observed in Figure 4 at the interface between sediments and water. This detail was not observed in the measurements of HHMs in sediments because the laboratories analyze the total concentrations, where $C_{d}$/$K_{d}$+ $C_{p}$, and the $C_{p}\;$ concentration predominates in the samples.

### 4.3. Model Assumptions, Limitations, and Ecological Implications

While CB served as a reference system to contextualize parameter ranges and model outputs, this study was not designed for site-specific application or empirical calibration. Rather, the model was developed to explore general physical processes governing HHM desorption and accumulation in estuarine sediments. Field data from CB, including reported sedimentation rates and Hg concentrations, were used qualitatively to guide parameter selection and verify that simulated $C_{bg}\;$ remained within empirically observed values, supporting the model’s realism under estuarine conditions.

The proposed 1D model was developed under the assumptions of continuous sedimentation without bottom erosion events. Technically, erosion could be easily included in the model; however, it may be difficult to control over extended periods of sediment dynamics. Changes in the porosity and tortuosity were also considered, which influence HHM transport and accumulation. These mechanisms may require a rheological model. Since the model operated under the assumption that the muddy substrate was not in motion, no assumption of the fluid type or the Newtonian fluid approximation is required.

A notable limitation of the current model is the assumption of a constant sedimentation velocity ($w_{g}$), whereas sedimentation processes in estuarine environments typically exhibit considerable spatial complexity. For instance, Figure 1 highlights sedimentation rates in CB varying by an order of magnitude between the Dique Channel mouth (10 m/25 y) and the northern bay sector (0.5 m/25 y). Such variations result from a combination of (a) bed load transport, (b) the precipitation of suspended particles, and (c) the flocculation of fine particles induced by brackish water salinity gradients. Determining the dominant mechanism among these and assessing the impact of wash load transport on sediment distribution throughout the bay remain challenging. Detailed geographic-specific analysis and further refinement of sedimentation mechanisms given in Equation (1) would thus enhance the model accuracy and applicability.

This modeling approach addresses a critical gap in the representation of sediment processes, particularly the understanding of $C_{bg}\;$ of HHMs in estuarine sediments, by integrating transport and reaction processes with site-specific hydro-sedimentary influences, often simplified in traditional frameworks. The relevance of $C_{bg\;}$ lies in its strong association with toxicological thresholds, bioavailability, and long-term ecological risks related to HHM pollution. Although a 1D framework offers notable advantages in computational efficiency, it limits horizontal transport and spatial interactions across estuarine gradients. Future initiatives could benefit from integrating diagenetic and hydrodynamic models to support evidence-based environmental management for preserving estuarine ecosystems.

## 5. Conclusions

Under physically valid assumptions, a novel 1D mathematical model was developed to simulate HHM dynamics in estuarine sediments, with broad applicability to water bodies influenced by HHM contamination. This numerical framework advances prior approaches by integrating coupled transport-reaction processes while dynamically accounting for porosity and tortuosity. Unlike conventional models, this approach includes molecular diffusion (*T*_1_), desorption (*T*_2_), sedimentation (*T*_3_), and water-turbulence exchange (*T*_4_) as a distinct method to estimate HHM $C_{bg}$. A notable innovation is the separation of $C_{d}$ and $C_{p}$, reaching an asymptotic equilibrium ($C_{d}$ = $C_{p}$ = $C_{bg}$) at the sediment base (*z* = 0). This mathematical formulation has not been previously reported in existing sediment models.

The $C_{bg}$ for Hg in CB ranged from 1.0 to 2.4 mg kg^−1^ dw, providing a valuable reference for future ecological risk assessments, pollution indexing, and numerical model calibration in estuarine sediments. $C_{bg}$ of Hg were characterized by very slow desorption. Particularly, $C_{bg}$ values did not remain constant but exhibited a drift, influenced by limited exchange with upper layers and overlying water. These findings may improve ecological risk assessment, environmental monitoring, and policy formulation to mitigate HHM impacts in CB and similar contaminated ecosystems.

Spatial and temporal variability in $C_{bg}$ arises from local sediment dynamics, precisely variations in precipitation rates, highlighting the need for zone-specific assessments within the same water body. Consequently, the 1D model can be applied to each node of the general hydrodynamic model of the basin.

The observed drift in $C_{bg}$ values demonstrates that profiling sediment layers dated with 14C does not necessarily reflect historical in situ concentrations, as reported by Fukue et al. [44]. This issue draws attention and stimulates future research using inverse models to restore HHM ancient profiles from in situ measurements.

The 1D model would be implemented as an interface between the water column and the consolidated substrate. This intermediate layer would capture processes at the sediment–water interface, transitioning from Newtonian fluid properties in the water–sediment upper layer of the bottom to solid substrate characteristics. The model acts as a universal boundary condition applicable across diverse aquatic systems receiving HHM contamination. However, site-specific calibration may be necessary due to local sedimentological and hydrodynamic conditions.

Future works will focus on integrating the 1D model into a 3D hydrodynamic framework to continuously simulate the long-term fate of sediments and HHMs to compare the model’s stratification predictions to in situ measurements of the vertical substrate. Such advancements could significantly aid in developing more effective management strategies to mitigate HHM pollution in coastal marine environments.

## Figures and Tables

**Table 1:** State variables and parameters used in this study.

Parameter	Description	Unit	Value	Reference
$C_{bg}$	HHM background concentration	g L^−1^, mg kg^−1^ (dw) *	see Table 2	calculated
$C_{D}$	Drag coefficient	/	2 × 10^−3^	[39]
$C_{d}$	Dissolved-phase HHM concentration	g L^−1^, mg kg^−1^ (dw) *	/	calculated
*C_m_*	Suspended-sediment mass concentration	g L^−1^	/	[40]
$C_{p}$	Particulate-phase HHM concentration	g L^−1^, mg kg^−1^ (dw) *	/	calculated
$C_{p0}$	Initial particulate HHM at precipitation	g L^−1^	/	assumed
$C_{v}$	Suspended-sediment volumetric concentration	/	10^−4^–10^−5^	assumed
*d*_50_	Median grain diameter of sediment	m	/	measured
*D*	Sediment thickness	m	0–1.6 **	calculated
*F*	Porosity–tortuosity factor	/	/	calculated
*HHMs*	Harmful heavy metals	g L^−1^, mg kg^−1^ (dw) *	varies	measured
$K_{d}$	Coefficient of equilibrium distribution	/	/	assumed
*m*	Exponent in the relationship of Sc and n	/	/	literature
*N*	Number of computational nodes	/	100	assumed
*n*	Porosity	/	0.4	[34]
*Q*	Molecular diffusion flux	kg m^−2^ s^−1^	varies	calculated
*S*	Salinity	/	0.06–35.7	assumed
$S_{C}$	Schmidt number	/	10–100	[6]
*t*	Time	s	0–8.64 × 10^8^ s	assumed
*T*_1_	Molecular diffusion rate	yr	0.3–3	calculated
*T*_2_	Desorption rate	yr	3.15 (for γ = 5 × 10^−8^)	calculated
*T*_3_	Sediment rate	yr	>31	calculated
*T*_4_	Turbulent exchange rate	yr	/	calculated
$u_{*}$	Friction (dynamic) velocity	m s^−1^	0–0.01	assumed
$w_{g}$	Settling velocity of sediments due to gravity	m s^−1^	10^−5^	assumed
*Y*	Dimensionless vertical coordinate	/	0–1	calculated
z	Vertical level within the substrate	m	0–1.6	calculated
$z_{0}$	Roughness parameter	m	/	literature
$\alpha_{S}$	Inverse Schmidt number $\left({Sc}^{-1}\right)$	/	0.01–0.1	[36]
*γ*	Desorption coefficient	s^−1^	5 × 10^−8^–1 × 10^−9^	[41]
∆y	Vertical grid size in dimensionless coordinates	/	1/(N − 1)	calculated
*θ*	Tortuosity	/	/	[35]
κ	Karman constant	/	0.41	literature
*ν*	Kinematic molecular viscosity of water	m^2^ s^−1^	10^−6^	constant
$\rho_{s}$	Sediment–particle density	kg m^−3^	2650	[39]
$\chi_{0}$	Molecular diffusion coefficient (water only)	m^2^ s^−1^	/	[17]
$\chi_{S}$	Molecular diffusion coefficients (with sediments)	m^2^ s^−1^	/	calculated

* $C_{d}$ and $C_{p}$ represent concentrations expressed in g L^−1^ or kg m^−3^ in the model for consistency, but in the figures, they are presented in mg kg^−1^ dry weight (dw) for easier comparison with laboratory data. Laboratories generally measure HHM concentrations in mg kg^−1^, dw. Note that this difference in units is important when interpreting model results and comparing them with laboratory data or figures. ** *D* (0–1.6 m) based on 28 years of sedimentation. “/” means no value.

**Table 2:** Model-derived Hg background concentrations ($C_{bg}$) at boundary (*z* = 0) under different simulation cases.

Case	Description	$\mathrm{Estimated}\;C_{bg}$ mg kg^−1^ (dw)	Observation
1	γ = 5 × 10^−8^ 1/s	1.4–1.7	Long-term equilibrium at *z* = 0
2	γ = 10^−8^ 1/s	1.0–1.2	Slower equilibrium from low γ
3	$C_{p0}$ increasing over 28 yr	2.0–2.4	Closest to observed CB field data
4	Variable sediment input *	2.0–2.2	Dynamic but consistent $C_{bg}\;$ at *z* = 0
-	Average Hg $C_{bg\;}$ (model)	0.2 ± 1.7	Variability across all cases

* Seasonal sediment variation using white noise flow 55–250 m^3^ s^−1^.

## Data Availability

Data available on request from the authors.

## Appendix A

Without the source term and molecular diffusion in a closed system, Equations (6) and (7) can be expressed as follows:

(A1) $$\frac{\partial C_{p}}{\partial t}=-\gamma \left(C_{p}-C_{d}\right)$$

(A2) $$\frac{\partial C_{d}}{\partial t}=\gamma \left(C_{p}-C_{d}\right),$$

This represents the desorption from the particulate to the dissolved form of the HHMs, with

$$\frac{\partial \left(C_{p}+C_{d}\right)}{\partial t}=0$$

Assuming the initial conditions $C_{p}\left(t=0\right)=C_{0}$ and $C_{d}\left(t=0\right)=0$, the respective analytical solution of the system (Equations (A1) and (A2)) becomes the following:

(A3) $$C_{p}\left(t\right)=\frac{C_{0}\left(1+e^{-2\gamma t}\right)}{2};\;C_{d}\left(t\right)=\frac{C_{0}\left(1-e^{-2\gamma t}\right)}{2},$$

until it reaches an equilibrium, where $C_{p}=C_{d}=\frac{C_{0}}{2}$ for t→∞. The characteristic scale of this process, given in Equation (4), according to Equation (A3) is $T_{2}=\frac{1}{2\gamma }.$

## Appendix B

To specify the boundary conditions for Equation (7) at the bottom surface, a flux equilibrium was established for the dissolved components of the HHMs:

(A4) $$-\bar{w^{\prime }C^{\prime }}+\nu \frac{\partial \bar{C}}{\partial z}=\alpha_{S}\nu \frac{\partial \bar{C_{d}}}{\partial z}$$

Here, the first and second terms on the left-hand side represent the turbulent and molecular fluxes of the HHMs with concentrations $\bar{C}$ in the water, respectively. The term on the right-hand side is the molecular diffusion flux of the substance in the sediments.

The sum of the turbulent and molecular fluxes was constant in a relatively thick layer, known as the near-surface bulk layer or “layer of constant fluxes”. This could be parameterized based on the K-theory of turbulence by defining the flux *q* within the logarithmic profile of the substance:

(A5) $$\;q=\frac{\left(\bar{C}-C_{d}\right)\kappa u_{*}}{ln(\frac{z}{z_{0}})},$$

where κ = Karman constant (κ=0.41); $u_{*}$ is the friction (dynamic) velocity in the near- surface layer; $z_{0}$ is the roughness parameter. In this case, *z* extends from the bottom surface $z_{0}$ upwards and is fixed with a reference value $\bar{C}$ of concentrations.

Introducing the drag coefficient $C_{D}$ and assuming that the concentration in water is $\bar{C}=0$ in Equation (A5), and based $\bar{C}\ll C_{d}$, Equation (A4) gives the following:

(A6) $$-C_{d}C_{D}^{\frac{1}{2}}u_{*}=\alpha_{S}\nu \frac{\partial \bar{C_{d}}}{\partial z},$$

which is Equation (8) when *A* = $C_{D}^{1/2}u_{*}$.

The fact that $\bar{C}\ll C_{d}$ in the water column was justified under the assumption that the volumetric sediment concentrations in the water were less than 10^−4^. In an extreme hypothetical scenario, where equilibrium is reached between the HHMs in the water column, encompassing both $C_{p}$ and $C_{d}$, the concentration $\bar{C}$ represents no more than 0.01% of the $C_{p}$.

## Appendix C

Generally, the molecular Schmidt number, *Sc*, characterizes the relationship between the molecular viscosity of water and the molecular diffusion of substances; it measures how fast the “diffusion of momentum” occurs relative to other fluid properties. Substances with a water temperature of approximately *Sc* = 10 can increase by one or two orders of magnitude [36].

The Schmidt number is associated with the porosity and tortuosity of a fluid composed of liquid and bottom sediments. Porosity and tortuosity are considered to play major roles in the increase of this number. In the proposed study by Maerki et al. [45], the molecular flow (2) was identified as follows:

(A7) $$Q=n\chi_{S}\frac{\partial C_{d}}{\partial z}=n\chi_{0}\theta^{-2}\frac{\partial C_{d}}{\partial z}={F^{-1}\chi }_{0}\frac{\partial C_{d}}{\partial z},$$

where *n* represents porosity, θ characterizes tortuosity, and *F* combines the effect of both; $\chi_{S}$ and $\chi_{0}$ are the molecular diffusion coefficients with and without sediment particles, respectively.

If $\chi_{0}=\nu$ and all diffusion effects (substance, porosity, and tortuosity) are assigned to the *Sc* number, then *Sc* = *F*. Based on Maerki et al. [45], we conclude the following:

(A8) $$Sc=1.02n^{-m}$$

where m > 1 (m = 1.81 in the cited work).

Consequently, the *Sc* value depends on the substrate level, residence time, and compaction of the sediment, among other factors. Shen and Chen [46] provided further details on the parameterizations of these effects in sediments.
